# Supplementary material for: Novel Physical Vapor Deposition Approach to Hybrid Perovskites: Growth of MAPbI3 Thin Films by RF-Magnetron Sputtering
Source: Sci Rep. 2018 Oct 18;8:15388. doi: 10.1038/s41598-018-33760-w (PMC6193984; doi:10.1038/s41598-018-33760-w)
Supplement: Supplementary file 1 — Supplementary Information [file 41598_2018_33760_MOESM1_ESM.docx]

Novel Physical Vapor Deposition Approach to Hybrid Perovskites: Growth of MAPbI_3_ Thin Films by RF-Magnetron Sputtering

Sara Bonomi,^a^ Daniela Marongiu,^b^ Nicola Sestu,^b^ Michele Saba,^b^ Maddalena Patrini,^c^ Giovanni Bongiovanni,^b^ Lorenzo Malavasi ^a,^*

SUPPLEMENTARY INFORMATION


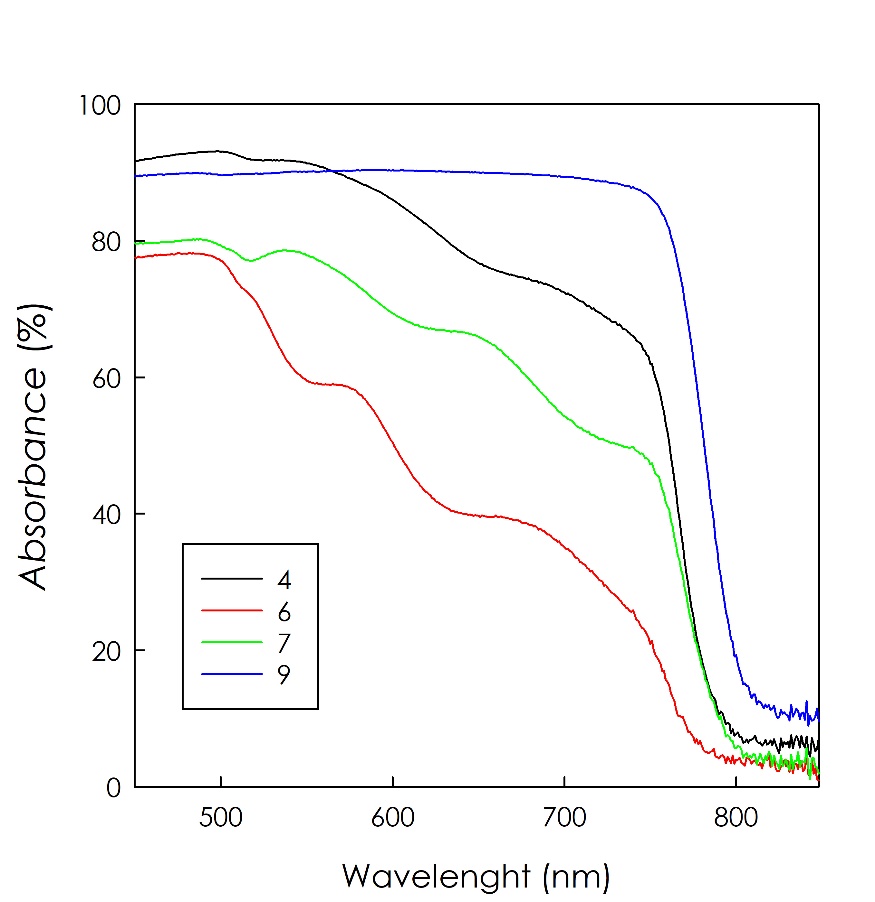


**Figure 1** UV-VIS transmittance spectrum of selected MAPI films.













**A**

**B**













**C**

**D**













**E**

**F**

**Figure 2** SEM images of MAPI films at two magnifications: film **6** (A at 20kX and B at 100kX), film **8** (C at 20kX and D at 100kX) and film **9** (E at 20kX and F at 100kX).

**c**

| Film | Thickness (nm) | FWHM peak at about 14° (°) |
| --- | --- | --- |
| 1 | < 200 | 0.26(1) |
| 2 | 220 | 0.227(5) |
| 3 | 300 | 0.241(3) |
| 4 | 350 | 0.341(4) |
| 5 | 440 | 0.264(3) |
| 6 | 510 | 0.165(3) |
| 7 | 640 | 0.185(3) |
| 8 | 940 | 0.147(3) |
| 9 | 3200 | 0.154(4) |

**Table 1** FWHM of the MAPI films considered in the present work.

**c**
